# An annotated fluorescence image dataset for training nuclear segmentation methods

**DOI:** 10.1038/s41597-020-00608-w

**Published:** 2020-08-11

**Authors:** Florian Kromp, Eva Bozsaky, Fikret Rifatbegovic, Lukas Fischer, Magdalena Ambros, Maria Berneder, Tamara Weiss, Daria Lazic, Wolfgang Dörr, Allan Hanbury, Klaus Beiske, Peter F. Ambros, Inge M. Ambros, Sabine Taschner-Mandl

**Affiliations:** 1 Tumor biology group, Children’s Cancer Research Institute, Zimmermannplatz 10, 1090 Vienna, Austria; 2 Labdia Labordiagnostik GmbH, Zimmermannplatz 8, 1090 Vienna, Austria; 3 Software Competence Center Hagenberg GmbH (SCCH), Softwarepark 21, 4232 Hagenberg, Austria; 4 ATRAB-Applied and Translational Radiobiology, Department of Radiation Oncology, Medical University of Vienna, Währinger Gürtel 18-20, 1090 Vienna, Austria; 5 Institute of Information Systems Engineering, TU Wien, Favoritenstrasse 9-11/194, 1040 Vienna, Austria; 6 Complexity Science Hub, Josefstädter Straße 39, 1080 Vienna, Austria; 7 Department of Pathology, Oslo University Hospital, Ullernchausséen 64, N-0379 Oslo, Norway; 8 Department of Pediatrics, Medical University of Vienna, Währinger Gürtel 18-20, 1090 Vienna, Austria

**Keywords:** Machine learning, Image processing

## Abstract

Fully-automated nuclear image segmentation is the prerequisite to ensure statistically significant, quantitative analyses of tissue preparations,applied in digital pathology or quantitative microscopy. The design of segmentation methods that work independently of the tissue type or preparation is complex, due to variations in nuclear morphology, staining intensity, cell density and nuclei aggregations. Machine learning-based segmentation methods can overcome these challenges, however high quality expert-annotated images are required for training. Currently, the limited number of annotated fluorescence image datasets publicly available do not cover a broad range of tissues and preparations. We present a comprehensive, annotated dataset including tightly aggregated nuclei of multiple tissues for the training of machine learning-based nuclear segmentation algorithms. The proposed dataset covers sample preparation methods frequently used in quantitative immunofluorescence microscopy. We demonstrate the heterogeneity of the dataset with respect to multiple parameters such as magnification, modality, signal-to-noise ratio and diagnosis. Based on a suggested split into training and test sets and additional single-nuclei expert annotations, machine learning-based image segmentation methods can be trained and evaluated.

## Abstract (summary)

**Table:** 

Measurement(s)	nucleus • Annotation • Frozen Section • Neuroblastoma • Touch Prep Slide • Centrifuged Smear Slide • cells grown on slide • Ganglioneuroblastoma • Wilms Tumor • HaCaT cell
Technology Type(s)	Fluorescence Imaging • machine learning
Factor Type(s)	nucleus segmentation
Sample Characteristic - Organism	Homo sapiens

Machine-accessible metadata file describing the reported data: 10.6084/m9.figshare.12570854

## Background & Summary

Bioimage analysis is of increasing importance in multiple domains including digital pathology, computational pathology, systems pathology or quantitative microscopy^1–6^. The field is currently rapidly expanding mainly facilitated by technological advances of imaging modalities in terms of resolution, throughput or automated sample handling. Moreover, publicly available databases and platforms allow the access to image datasets and annotations enabling the research community to develop sophisticated algorithms for complex bioimage analysis.

Digital pathology relies on tissue sections as a basis for diagnosing disease type and grade or stage^7,8^. Moreover, the accurate quantification of subcellular information (including nuclear features) at the single cell level is critical for the characterization of tumor heterogeneity, plasticity, response to therapeutic intervention^9,10^, and others. Several approaches to visualise subcellular compartments such as nuclear morphology and/or biological markers in tissues or cell preparations are well established: Hematoxylin and eosin (H&E) histological stainings, immunohistochemical (IHC) and immunofluorescence (IF) stainings. Whereas H&E and IHC stainings and visualisation by bright field microscopy are standard procedures in routine diagnostic laboratories and pathology departments, IF is more often employed in research settings than in routine diagnostics. Prominent examples recently proved the feasibility and power of IF-based quantitative analysis for e.g. detection of blood circulating or bone marrow disseminated tumor cells for minimal residual disease (MRD) diagnostics or the detection of genetic alterations by fluorescence *in situ* hybridization (FISH)^11–13^. Although the digitization of fluorescence stainings is more challenging and time consuming when compared to the digitization of H&E or IHC stainings, up to 90 or more cellular or sub-cellular markers can be visualized in multiplex assays^14,15^. This depicts a beneficial gain of information on individual cells and cell compartments.

A prerequisite for any reliable quantitative image-based analysis, however, is the accurate segmentation of nuclei in fluorescence images^5,6,16^. In order to reach sufficient power for statistical analysis, fully automated segmentation algorithms are preferred. While tissues and cell preparations presenting with well separated nuclei can be segmented based on simple intensity thresholds, densely packed tissues or cell aggregations require more sophisticated algorithms for nuclear segmentation. Segmentation algorithms focusing on the separation of objects (instances) are called instance segmentation algorithms or instance aware segmentation algorithms. Although designed to split aggregating nuclei, they frequently fail to separate each nucleus in cases of tightly clustered nuclei^17^. Remaining aggregations of nuclei lead, in the worst case, to their exclusion from the downstream analysis potentially causing a biologically significant bias.

To overcome these drawbacks, novel deep learning-based image segmentation approaches are currently developed in many labs worldwide. They promise to solve most segmentation problems as long as high-quality expert-annotated datasets are available to train the systems. However, there are only a limited number of annotated nuclear image datasets publicly available. While annotated datasets outlining nuclei in H&E or IHC images can be obtained, a comprehensive dataset including IF images of tissue sections of diverse origin and annotated nuclei is currently not available, to the best of our knowledge. Apart from the tedious process of annotation, this may be due to the fact that it is challenging to decide whether an aggregation consists of one or multiple nuclei. This is because in epifluoresecence microscopy images, nuclei often appear blurry and within cell aggregations their borders are frequently not definable.

In summary, the time consuming and challenging process of annotation hampered the generation and publication of annotated fluorescence image datasets including tightly aggregated and overlapping nuclei.

We hereby present an expert-annotated comprehensive dataset^18^ that can be used to train machine learning-based nuclear segmentation algorithms. The presence and annotation of tightly aggregating and partially overlapping nuclei enable the algorithms to learn how to solve the task of accurate instance aware nuclear segmentation. The dataset consists of 79 annotated IF images of different biological tissues and cells of pathological and non-pathological origin covering the main preparation types used in imaging-based biomedical research settings: Schwann cell stroma-rich tissue (from a ganglioneuroblastoma) cryosections, a Wilms tumor tissue cryosection, a neuroblastoma tumor tissue cryosection, bone marrow cytospin preparations infiltrated with neuroblastoma cells, neuroblastoma tumor touch imprints, cells of two neuroblastoma cell lines (CLB-Ma, STA-NB10) cytospinned on microscopy glass slides and cells of a normal human keratinocyte cell line (HaCaT) cytospinned or grown on microscopy glass slides. The characteristics of neuroblastoma, and the Schwann cell stroma-rich ganglioneuroblastoma tumors have been previously described by Shimada et al. in detail^19^. Multiple modalities (Zeiss Axioplan II, Zeiss- and Leica laser scanning microscopes (LSM)) were used for image acquisition while using different magnifications (10x, 20x, 40x, 63x objectives). Nuclei in IF images were annotated by trained biologists, carefully curated by an experienced disease expert and finally reviewed and curated by an external disease expert and pathologist. The final annotated dataset, forming the ground truth dataset, was split into a training set and a test set to be used for machine learning-based image segmentation architectures. The training set consists of images with similar characteristics while the test set partially consists of images with varying image characteristics. To enable a detailed assessment of the generalizability of trained segmentation algorithms with respect to image parameters such as the magnification or the signal-to-noise ratio, the images of the test set were divided into 10 classes based on common image characteristics. In addition to the expert-annotated ground truth, randomly selected nuclei from images of each class were marked on the raw images and presented to two independent experts subject to annotation. These annotations, further called single-nuclei annotations, serve to 1. validate the quality of the annotated dataset by comparing the ground truth annotations to the single-nuclei expert annotations and 2. to set a baseline for machine learning-based image segmentation architectures.

In conclusion, the proposed expert-annotated dataset presents a heterogeneous, real-world dataset consisting of fluorescence images of nuclei of commonly used tissue preparations showing varying imaging conditions, sampled using different magnifications and modalities. The dataset can be used to train and evaluate machine learning-based nuclear image segmentation architectures, thereby challenging their ability to segment each instance of partially highly agglomerated nuclei.

## Methods

### Patient samples

Tumor ($n$ = 4) and bone marrow ($n$ = 4) samples of stage M neuroblastoma patients, the Schwann cell stroma-rich part of a patient with a ganglioneuroblastoma tumor and one wilms tumor patient were obtained from the Children’s Cancer Research Institute (CCRI) biobank (EK.1853/2016) within the scope of ongoing research projects. In addition, two patient-derived neuroblastoma cell lines (CLB-Ma, STA-NB10) were used. Written informed consent has been obtained from patients or patient representatives. Ethical approval for IF staining and imaging was obtained from the ethics commission of the Medical University of Vienna (EK1216/2018). All authors confirm that we have complied with all relevant ethical regulations.

### Preparation and IF-staining of tumor tissue cryosections

The fresh-frozen tumor tissues of one ganglioneuroblastoma patient, one neuroblastoma patient and one Wilms tumor patient were embedded into tissue-tek-OCT and 4 $\mu m$ thick cryosections were prepared. Sections were mounted on Histobond glass slides (Marienfeld), fixed in 4.5% formaledhyde and stained with 4,6-diamino-2-phenylindole (DAPI), a blue fluorescent dye conventionally used for staining of nuclei for cellular imaging techniques. Finally, slides were covered with Vectashield and coverslips were sealed on the slides with rubber cement.

### Preparation and IF-staining of HaCaT human skin keratinocyte cell line

The HaCaT cell line, a spontaneously transformed human epithelial cell line from adult skin^20^, was cultivated either in culture flasks or on microscopy glass slides. Cell cultures were irradiated (2 and 6 Gy), harvested, cytospinned, air-dried and IF stained. Cells grown and irradiated on the glass slides were directly subjected to IF staining. Cells were fixed in 4% formaldehyde for 10 minutes at 4 °C, and were permeabilized with 0.1% sodium dodecyl sulfate (SDS) in PBS for 6 minutes. Slides were mounted with antifade solution Vectashield containing DAPI and coverslips were sealed on the slides with rubber cement.

### Preparation and IF-staining of tumor touch imprints and bone marrow cytospin preparations

Touch imprints were prepared from fresh primary tumors of 4 stage M neuroblastoma patients as previously described^21^. Mononuclear cells were isolated from bone marrow aspirates of 3 stage M neuroblastoma patients by density gradient centrifugation and cytospinned as described^22^. After fixation in 3.7% formaldehyde for 3 minutes, cells were treated according to the Telomere PNA FISH Kit Cy3 protocol (Dako), mounted with Vectashield containing DAPI, covered and sealed.

### Preparation and IF-staining of neuroblastoma cell line cytospin preparations

STA-NB-10 and CLB-Ma are cell lines derived from neuroblastoma tumor tissue of patients with stage M disease. Preparation and drug-treatment were conducted as described^23^. Briefly, cells were cultured in the absence or presence of 5 nM topotecan, a chemotherapeutic drug for 3 weeks, detached and cytospinned to microscopy glass slides. Preparations were air-dried, fixed in 3.7% formaldehyde, immuno- and DAPI stained, covered and sealed.

### Fluorescence imaging

Samples were imaged using 1. an Axioplan-II microscope from Zeiss equipped with a Maerzhaeuser slide scanning stage and a Metasystems Coolcube 1 camera using the Metafer software system (V3.8.6) from Metasystems, 2. an Axioplan-II microscope from Zeiss equipped with a Zeiss AxioCam Mrm 1 using the Metasystems ISIS Software for microscopy image acquisition, 3. an LSM 780 microscope from Zeiss equipped with an Argonlaser 458 nm, a photomultiplier tube (PMT) detector (371–740 nm) and a motorized Piezo Z-stage using the Zeiss Zen software package and 4. a SP8X from Leica equipped with a Diode Laser and a PMT detector (447–468 nm). For the presented dataset, we digitized the DAPI staining pattern representing nuclear DNA. Additional immunofluorescence or FISH stainings were in part available. An automatic illumination time was set as measured by pixel saturation (Metasystems Metafer and ISIS) or defined manually (Zeiss and Leica LSMs). Objectives used were a Zeiss Plan-Apochromat 10$\times$ objective (Zeiss Axioplan II; numerical aperture 0.45; air), a Zeiss 20$\times$ Plan-Apochromat (Zeiss LSM 780; numerical aperture 0.8; oil), a Zeiss 20$\times$ Plan-Apochromat (Leica SP8X; nuermical aperture 0.75; oil), a Zeiss Plan-Neofluar 40$\times$ objective (Zeiss Axioplan II; numerical aperture 0.75) and a Zeiss Plan-Apochromat 63$\times$ objective (Zeiss Axioplan II, Zeiss LSM 780 and Leica SP8X; numerical aperture 1.4; oil). Representative field of views (FOVs) were selected according to the following quality criteria: sharpness, intact nuclei and a sufficient number of nuclei.

### Ground truth annotation

**Fig. 1:**
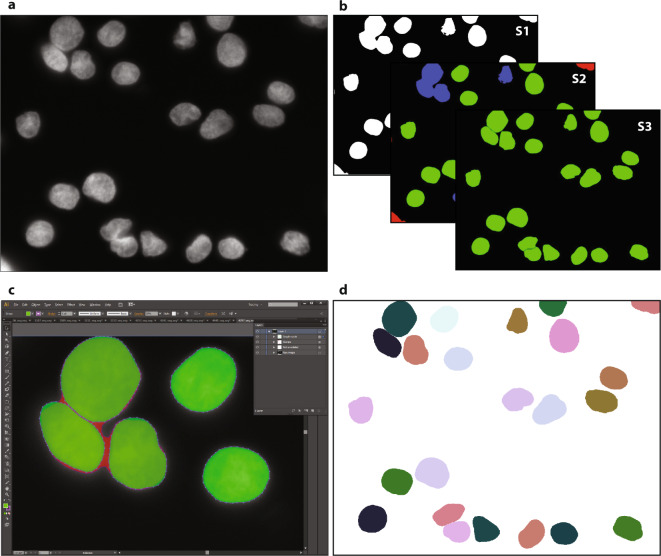
Workflow for ground truth image annotation. (**a**) Raw image visualizing HaCaT cytospinned nuclei. (**b**) A machine learning framework was used to annotate the raw image, learning from user interaction within three consecutive steps: S1. foreground extraction, S2. connected component classification (red = non-usable objects, blue = nuclei aggregations, green = single nuclei) and S3. splitting of aggregated objects into single nuclei, resulting in an annotation mask. (**c**) Zoom-in of the SVG-file showing the nuclear image overlaid with polygons representing each annotated nucleus. Polygons were modified by expert biologists to fit effective nuclear borders. Challenging decisions on how to annotate nuclei, mainly occurring due to aggregated or overlapped nuclei, were presented to an expert pathologist and corrected to obtain the final ground truth. (**d**) The curated SVG-file was transformed into a labeled nuclear mask.

Nuclei image annotation was performed by students and expert biologists trained by a disease expert. To accelerate the time consuming process of image annotation, a machine learning-based framework (MLF) was utilized supporting the process of annotation by learning characteristics of annotation in multiple steps^24^. The MLF annotations result in a coarse annotation of nuclear contours and have to be refined to serve as ground truth annotation. Therefore, annotated images were exported as support vector graphic (SVG) files and imported into Adobe Illustrator (AI) CS6. AI enables the visualization of annotated nuclei as polygons overlaid on the raw nuclear image and provides tools to refine the contours of each nucleus. An expert biologist and disease expert carefully curated all images by refining polygonal contours and by removing polygons or adding them, if missing. Finally, an expert pathologist was consulted to revise all image annotations and annotations were curated according to the pathologist’s suggestions. In cases where decision finding was difficult, a majority vote including all experts’ suggestions was considered and annotations were corrected accordingly. Images were exported and converted to Tagged Image File Format (TIFF) files, serving as nuclear masks in the ground truth dataset. A sample workflow is illustrated in Fig. 1.

### Dataset split

As the dataset is intended to be used to train and evaluate machine learning-based image segmentation methods, we created a dataset split into training set and test set. The training set consists of multiple images of ganglioneuroblastoma tissue sections, normal cells (HaCaT) as cytospin preparations or grown on slide, and neuroblastoma tumor touch imprints and bone marrow preparations. For each of these types of preparation, multiple images using the same magnification (20x or 63x) imaged with the same modality (Zeiss Axioplan II and the Metasystems Metafer Software) and showing a good signal-to-noise ratio were included. The test set consists of additional images of these preparation types and moreover, includes images of different preparation types (e.g. neuroblastoma cell line preparations, Wilms tumor and neuroblastoma tumor tissue sections) imaged with different modalities (Zeiss and Leica confocal LSM; Zeiss and Metafer ISIS software) and different signal-to-noise ratios.

To enable an objective comparison of image segmentation architectures to the ground truth annotations with respect to varying imaging conditions, we classified each image of the test set into one of 10 classes, according to criteria such as sample preparation, diagnosis, modality and signal-to-noise ratio. The details are presented in Table 1. The recommended dataset split into training set and test set and the test set classes can be downloaded along with the dataset.

**Table 1:** Test set split into 10 classes to evaluate the generalizability of machine learning-based image segmentation methods with respect to varying imaging conditions.

Acronym	Description
GNB-I	ganglioneuroblastoma tissue sections
GNB-II	ganglioneuroblastoma tissue sections with a low signal-to-noise ratio
NB-I	neuroblastoma bone marrow cytospin preparations
NB-II	neuroblastoma cell line preparations imaged with different magnifications
NB-III	neuroblastoma cell line preparations imaged with LSM modalities
NB-IV	neuroblastoma tumor touch imprints
NC-I	normal cells cytospin preparations
NC-II	normal cells cytospin preparations with low signal-to-noise ratio
NC-III	normal cells grown on slide
TS	other tissue sections (neuroblastoma, Wilms)

### Single-nuclei annotation

To set a baseline for machine learning-based image segmentation methods and to validate the proposed dataset, 25 nuclei were randomly sampled from the ground truth annotations for each of the classes, marked on the raw images and presented to two independent experts for image annotation. Annotation was carried out by a biology expert with long-standing experience in nuclear image annotation, further called annotation expert, and a biologist with experience in cell morphology and microscopy, further called expert biologist. Nuclei were annotated using SVG-files and Adobe illustrator. The single-nuclei annotations, described as single-cell annotations within the dataset, can be downloaded along with the dataset.

### Annotation criteria

The annotation of nuclei in tissue sections or tumor touch imprints is challenging and may not be unambiguous due to out-of-focus light or nuclei, damaged nuclei or nuclei presenting with modified morphology due to the slide preparation procedure. We defined the following criteria to annotate nuclear images:

- Only intact nuclei are annotated, even if the nuclear intensity is low in comparison to all other nuclei present.
- Nuclei have to be in focus.
- If parts of a nucleus are out of focus, only the part of the nucleus being in focus is annotated.
- Nuclear borders have to be annotated as exact as resolution and blurring allows for.
- Nuclei are not annotated if their morphology was heavily changed due to the preparation procedure.
- Nuclei from dividing cells are annotated as one nucleus unless clear borders can be distinguished between the resulting new nuclei.

## Data Records

**Fig. 2:**
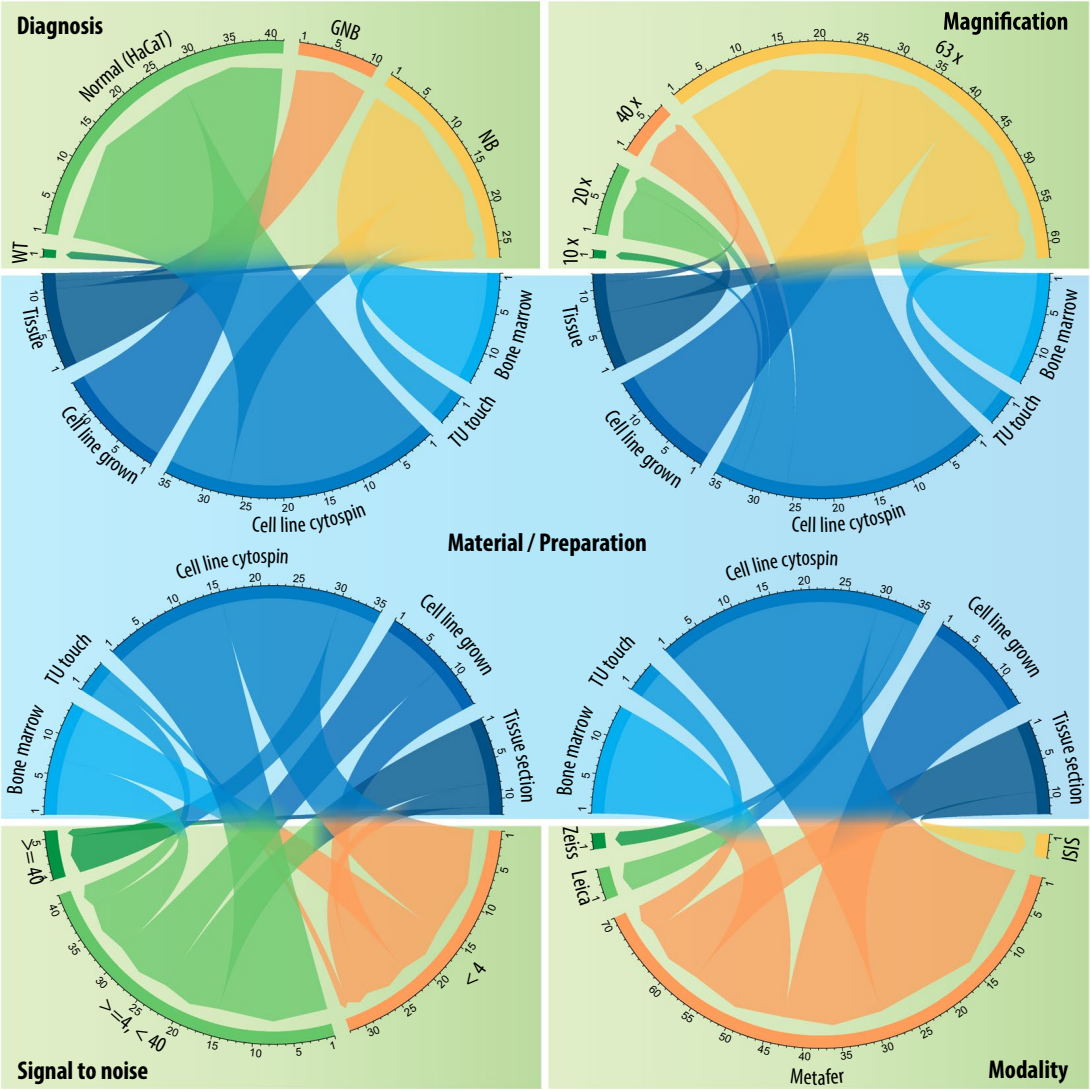
Heterogeneity of the proposed dataset with respect to the type of preparation. GNB: ganglioneuroblastoma, NB: neuroblastoma, TU touch: tumor touch imprint, Tissue: tissue section.

The dataset presented here is hosted in the BioStudies database under accession number S-BSST265 (https://identifiers.org/biostudies:S-BSST265)^18^. It consists of 79 fluorescence images of immuno and DAPI stained samples containing 7813 nuclei in total. The images are derived from one Schwann cell stroma-rich tissue (from a ganglioneuroblastoma) cryosection (10 images/2773 nuclei), seven neuroblastoma (NB) patients (19 images/931 nuclei), one Wilms patient (1 image/102 nuclei), two NB cell lines (CLB-Ma, STA-NB10) (8 images/1785 nuclei) and a human keratinocyte cell line (HaCaT) (41 images/2222 nuclei). In addition, the dataset is heterogenous in aspects such as type of preparation, imaging modality, magnification, signal-to-noise ratio and other technical aspects. A summary of the dataset composition and relevant parameters, e.g. diagnosis, magnification, signal-to-noise ratio and modality with respect to the type of preparation is presented in Fig. 2. Detailed information for each image is provided along with the dataset. The signal-to-noise ratio was calculated as follows. We used the binarized ground truth annotation masks to calculate the mean-foreground (nuclear) and mean-background signal. First, we calculated the mean intensity of all raw image pixels covered by the masks’ foreground region, resulting in the mean-foreground signal. By applying a morphological dilation on the binary foreground region using a disk-shaped structuring element of size 30 pixels and by inverting the resulting mask, mean intensity values of raw image pixels covered by the inverted mask were calculated, resulting in the mean-background signal. Dilation was applied to exclude pixels neighboring nuclei from the calculation as they do not represent the background signal, but present increased intensity values due to blurred nuclei.

Each image of the dataset is provided in TIFF-format. In addition, we provide two types of annotations: annotated, labeled masks in TIFF-format and SVG-files describing the exact position of each nucleus as a polygon. The SVG-files reference a nuclear image, stored in Joint Photographic Experts Group (JPEG)-format, and provide all annotated objects within an additional layer.

## Technical Validation

To validate the proposed dataset and to set a baseline for machine-learning based image segmentation methods, we compared the single-nuclei annotations to the ground truth annotation. We calculated the Dice coefficient $DC=\frac{X\cap Y}{|X\,|+|\,Y|}$ of each nucleus comparing the ground truth annotation $X$ and the corresponding expert annotation $Y$. Finally, we computed the mean Dice coefficient for each of the classes by calculating the mean value of the Dice coefficients of all annotations of a class. The overall Dice coefficient was computed by calculating the mean value of all annotations of all classes. The results are presented in Table 2.

**Table 2:** Mean Dice coefficient between randomly selected nuclei of the manual annotations and the ground truth annotations with respect to the human annotator.

Annotator	GNB-I	GNB-II	NB-I	NB-II	NB-III	NB-IV	NC-I	NC-II	NC-III	TS	Overall
Biol. exp.	**0.853**	**0.781**	**0.883**	0.932	**0.802**	**0.946**	**0.969**	**0.869**	**0.969**	**0.895**	**0.890**
Annot. exp.	0.877	0.849	0.896	**0.892**	0.888	0.957	**0.969**	0.928	0.973	**0.895**	0.912

Annot. exp.: annotation expert, Biol. exp.: expert biologist, GNB-I: ganglioneuroblastoma tissue sections, GNB-II: ganglioneuroblastoma tissue sections with a low signal-to-noise ratio, NB-I: neuroblastoma bone marrow cytospin preparations, NB-II: neuroblastoma cell line preparations with different magnifications, NB-III: neuroblastoma cell line preparations with different magnifications and imaged with LSM modalities, NB-IV: neuroblastoma tumor touch imprints, NC-I: normal cells (HaCaT) cytospin preparations, NC-II: normal cells (HaCaT) with low signal-to-noise ratio, NC-III: normal cells (HaCaT) grown on slide, TS: other tissue sections (neuroblastoma, Wilms tumor). Bold values set the baseline for machine learning-based image segmentation methods.

The overall Dice coefficients of 0.890, achieved by the expert biologist, and 0.912, achieved by the annotation expert, show that the concordance in annotations of nuclear contours on the pixel level is high between expert annotations and the ground truth. A Dice score close to 1 (the optimal score) cannot be achieved due to the nature of fluorescence images (e.g. blurriness resulting in fuzzy nuclear borders) and highly overlapping nuclei with partially invisible borders. Varying Dice coefficients between classes are due to different imaging conditions. For example, images in the GNB-II class were more blurry and present lower signal-to-noise ratios than images of e.g. the NC-I to NC-III classes, thus exact nuclear contours cannot be determined. Dice scores achieved by the annotation expert were higher than the scores achieved by the expert biologist in eight out of ten classes. This was an expected result, as the task of image annotation differs from image acquisition and visual biological interpretation. Thus, the annotation expert’s experience in image annotation was of benefit to achieve higher coefficients. The presented scores and the released single-nuclei ground truth can be further used to benchmark the accuracy of machine learning-based image segmentation architectures in comparison to the baseline set by human annotators.

## Data Availability

We provide code to transform predicted annotation masks in TIFF-format to SVG-files for curation by experts as well as the transformation from SVG-files to TIFF-files. The contour sampling rate when transforming mask objects to SVG-descriptions can be set in accordance to the size of predicted nuclei. Therefore, new nuclei image annotation datasets can easily be created utilizing the proposed framework and a tool to modify SVG-objects, such as Adobe Illustrator. The code is written in python and is publicly available under https://github.com/perlfloccri/NuclearSegmentationPipeline.
